# Supplementary material for: Fatty Acid Synthesis and Degradation Interplay to Regulate the Oxidative Stress in Cancer Cells
Source: Int J Mol Sci. 2019 Mar 18;20(6):1348. doi: 10.3390/ijms20061348 (PMC6471536; doi:10.3390/ijms20061348)
Supplement: Supplementary file 1 [file ijms-20-01348-s001.pdf]

Supplementary material

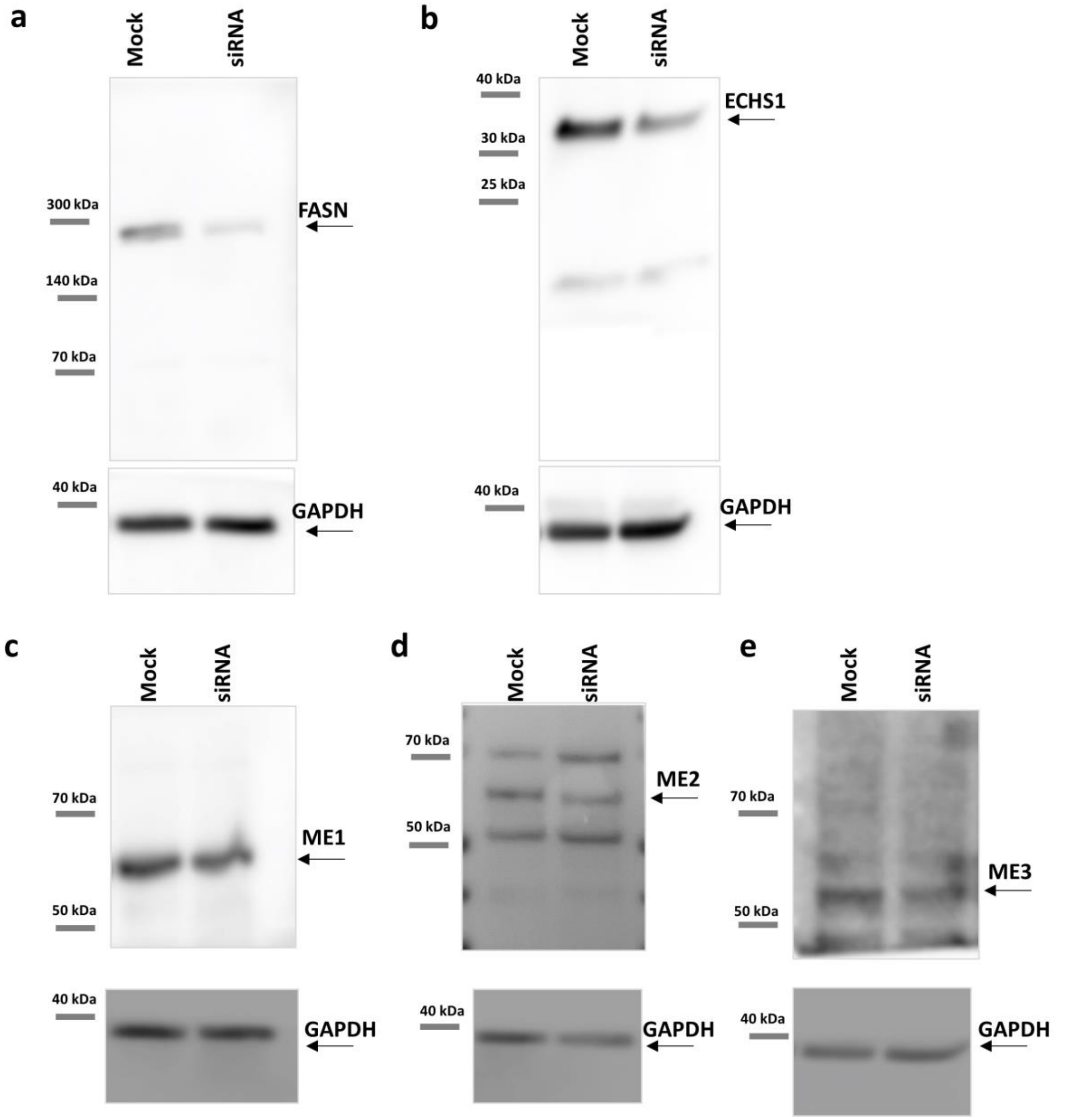

Supplementary Fig. S1 Western blots showing the effects of the FASN, ECHS1, ME1, ME2, ME3 siRNAs on the protein levels of their targets. The level of GAPDH is used as a control.

a

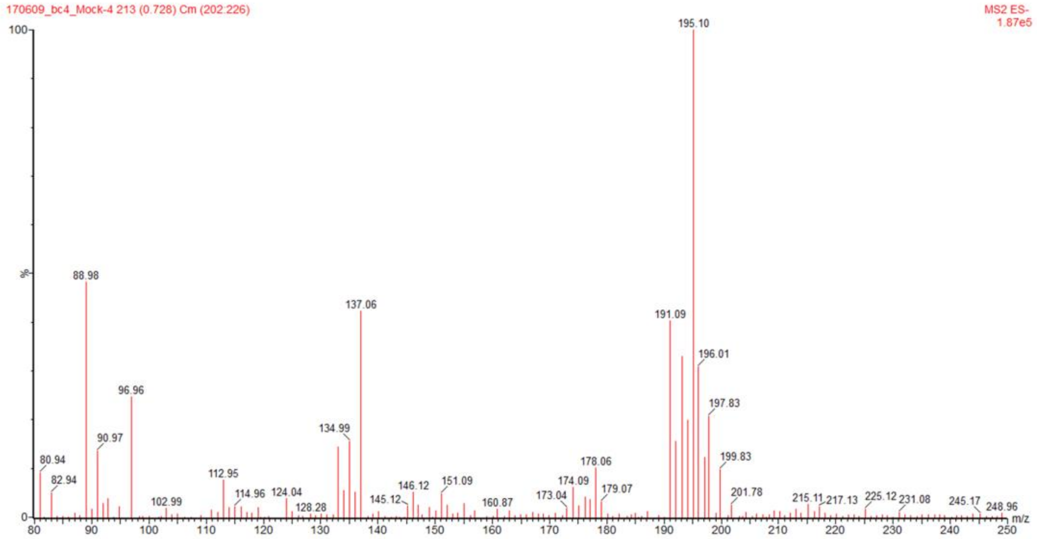
170609_bc4_Mock-4 213 (0.728) Cm (202:226)
MS2 ES-
1.87e5
100
%
0
195.10
88.98
137.06
191.09
196.01
197.83
96.96
90.97
134.99
80.94
82.94
178.06
199.83
112.95
146.12
151.09
174.09
114.96
124.04
179.07
102.99
128.28
145.12
160.87
173.04
201.78
215.11
217.13
225.12
231.08
245.17
248.96
m/z
80
90
100
110
120
130
140
150
160
170
180
190
200
210
220
230
240
250

a’

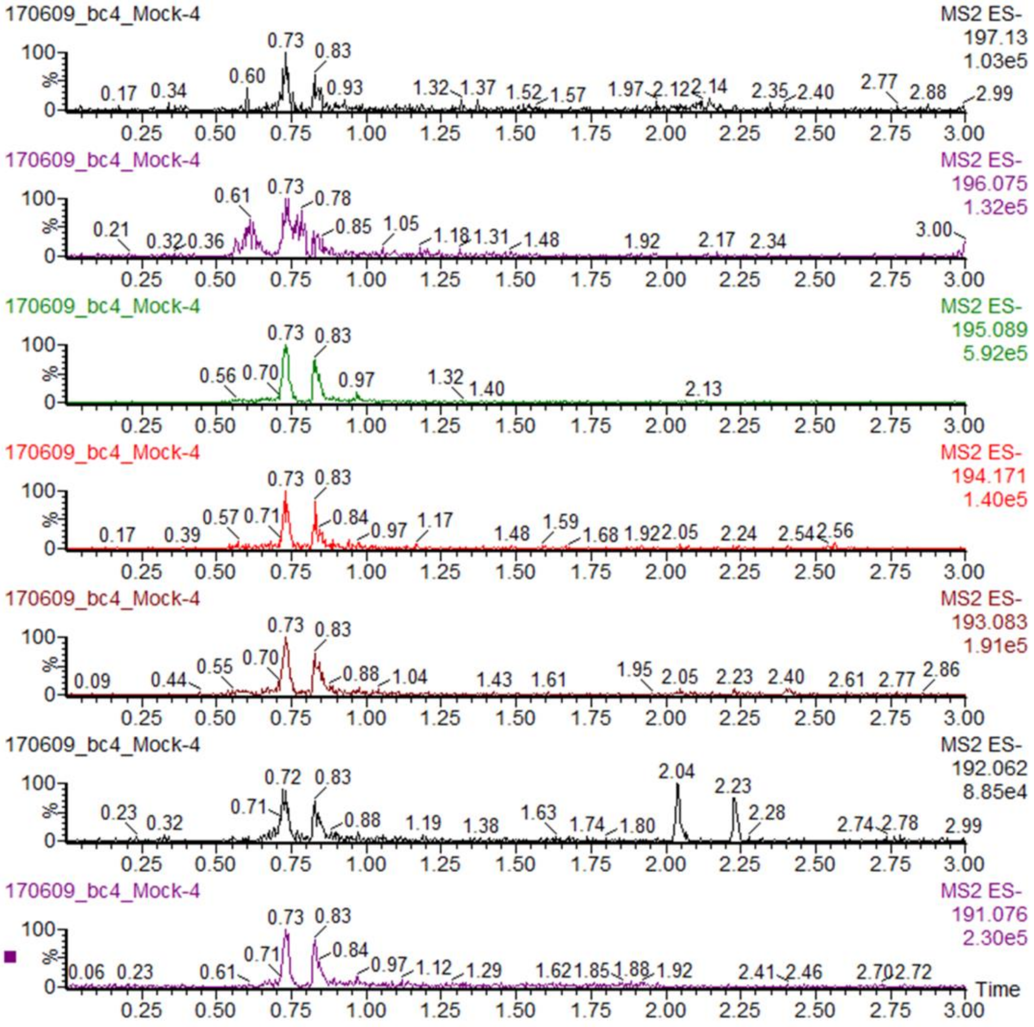
170609_bc4_Mock-4
MS2 ES-
197.13
1.03e5
170609_bc4_Mock-4
MS2 ES-
196.075
1.32e5
170609_bc4_Mock-4
MS2 ES-
195.089
5.92e5
170609_bc4_Mock-4
MS2 ES-
194.171
1.40e5
170609_bc4_Mock-4
MS2 ES-
193.083
1.91e5
170609_bc4_Mock-4
MS2 ES-
192.062
8.85e4
170609_bc4_Mock-4
MS2 ES-
191.076
2.30e5
100
%
0
Time
0.25
0.50
0.75
1.00
1.25
1.50
1.75
2.00
2.25
2.50
2.75
3.00

b

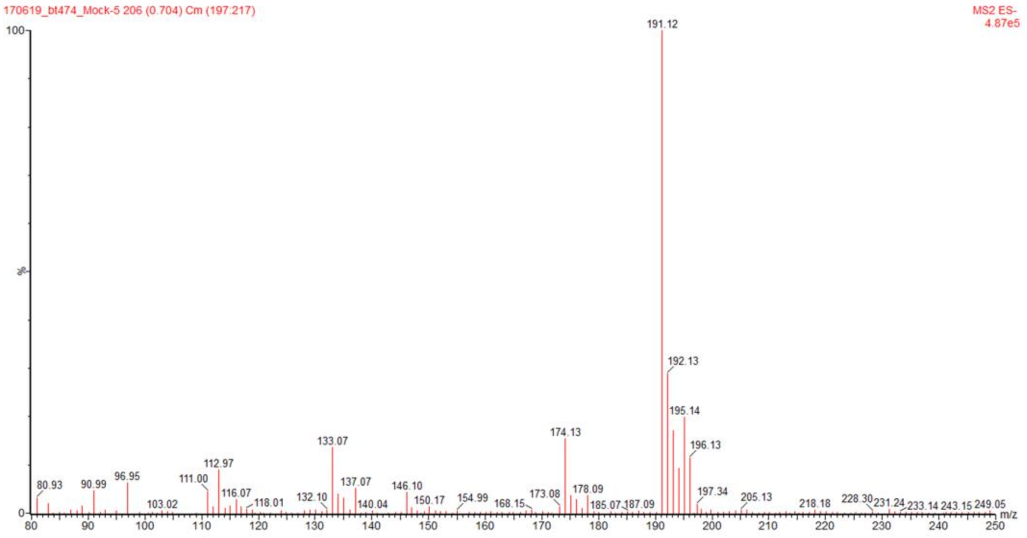

170619_bt474_Mock-5 206 (0.704) Cm (197:217)
MS2 ES-
4.87e5
191.12
192.13
195.14
196.13
174.13
133.07
112.97
80.93
90.99
96.95
111.00
116.07
103.02
118.01
132.10
137.07
140.04
146.10
150.17
154.99
168.15
173.08
178.09
185.07
187.09
197.34
205.13
218.18
228.30
231.24
233.14
243.15
249.05
m/z
%
100
0

b’

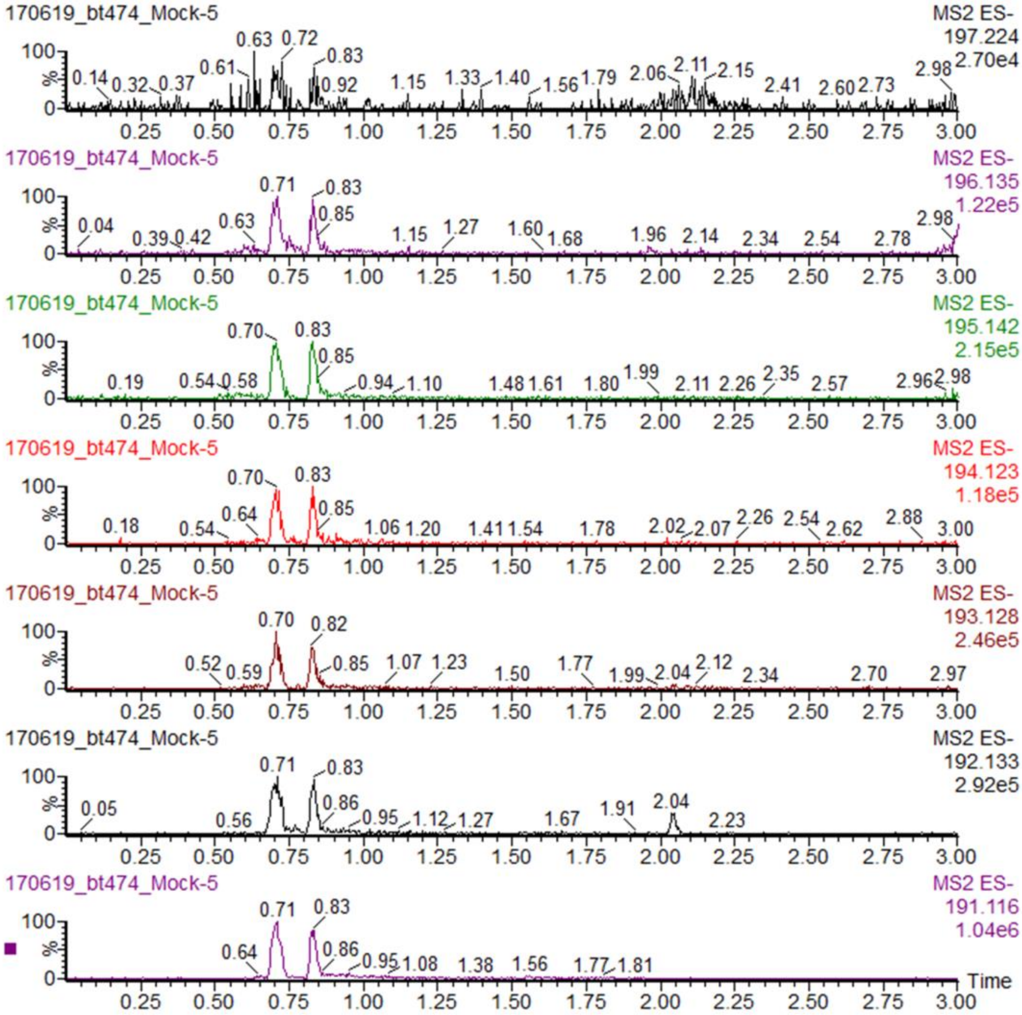

170619_bt474_Mock-5
MS2 ES-
197.224
2.70e4
170619_bt474_Mock-5
MS2 ES-
196.135
1.22e5
170619_bt474_Mock-5
MS2 ES-
195.142
2.15e5
170619_bt474_Mock-5
MS2 ES-
194.123
1.18e5
170619_bt474_Mock-5
MS2 ES-
193.128
2.46e5
170619_bt474_Mock-5
MS2 ES-
192.133
2.92e5
170619_bt474_Mock-5
MS2 ES-
191.116
1.04e6
Time
%

c

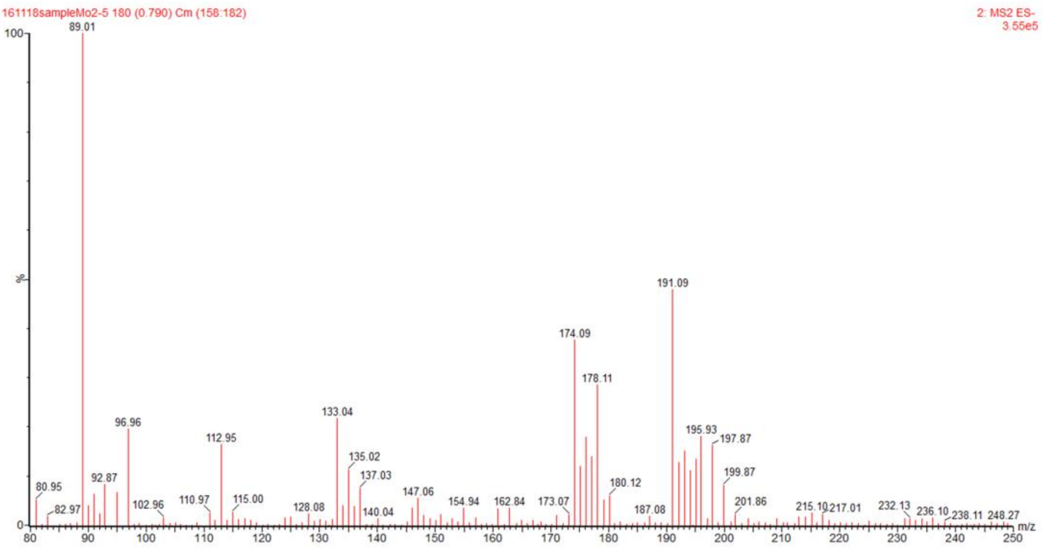

c’

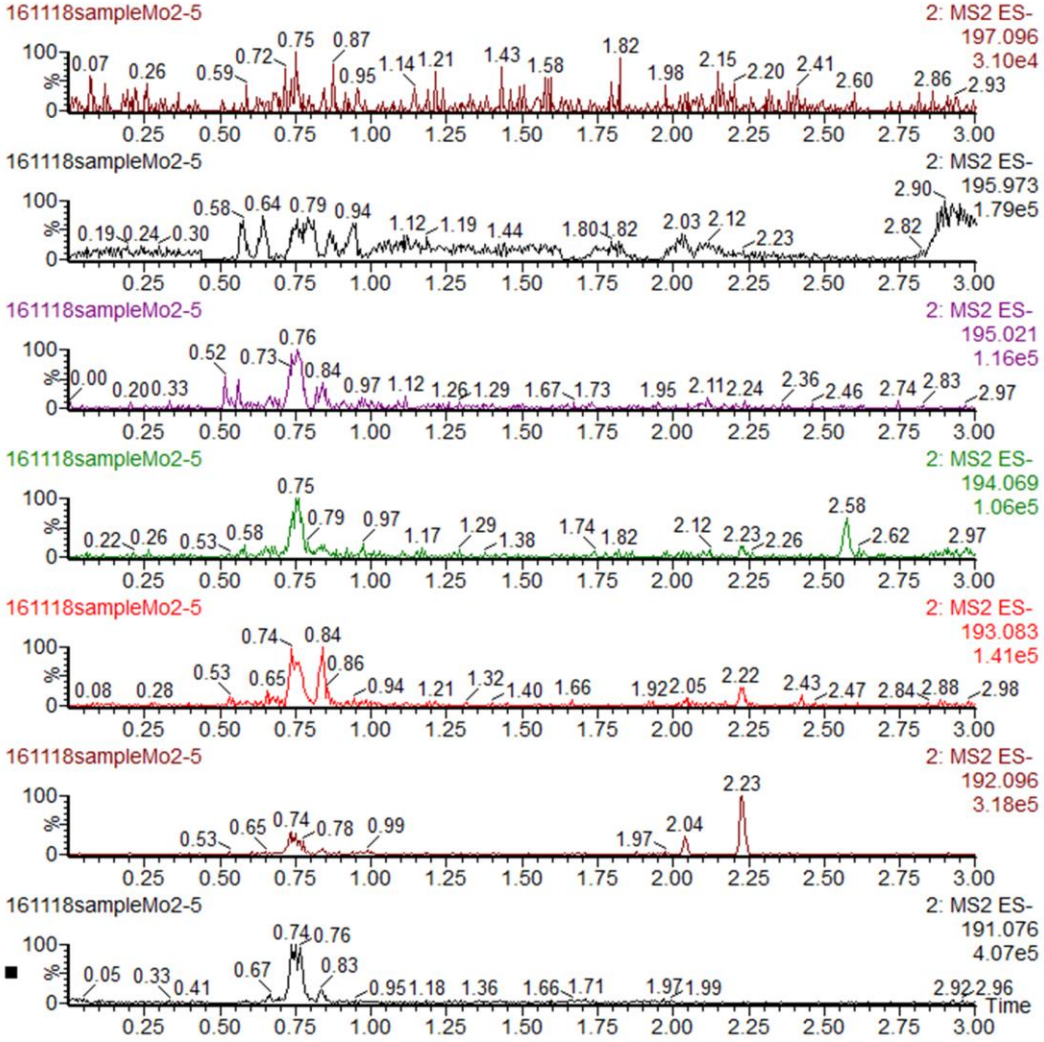

Supplementary Fig. S2 Ms spectra (a,b,c) and extracted ion chromatograms (a’,b’,c’) of citrate M0-M6 isotopomers in BCC (a,a’), BT-474 (b,b’) and MCF7 (c,c’) cells. Citrate retention time 0.7-0.75 min.

Supplementary methods

System identification

Our system consists on a set of inputs (6 independent reaction rates), noted as x_i and 10 independent measurable outputs (the fractions of M1 to M4 malate and the fractions M1 to M6 citrate), noted as y_i. The Jacobian Matrix, which can be numerically calculated using the EMU framework, provides the relation between changes in the input values and the changes in the values of measurable outputs.

$$J = \begin{pmatrix} \frac{\partial y_1}{\partial x_1} & \cdots & \frac{\partial y_1}{\partial x_6} \\ \cdots & \cdots & \cdots \\ \frac{\partial y_{10}}{\partial x_1} & \cdots & \frac{\partial y_{10}}{\partial x_6} \end{pmatrix} \quad [1]$$

A change in the inputs modifies the observable outputs in the following way:

$$d\vec{y} = Jd\vec{x} \quad [2]$$

If some of the columns of the Jacobian matrix are linearly dependent, its Kernel or null space has one or more dimensions and the inputs to the system could be modified in such a way that the measurable outputs remain unchanged.

$$d\vec{x} \in Ker(J) => Jd\vec{x} = \vec{0} \quad [3]$$

Therefore, many different sets of inputs would lead to the same measurable outputs and the system remain unidentifiable.

Singular value decomposition of the Jacobian matrix expresses it as the product of two unitary matrixes U and V and a rectangular diagonal matrix ∑, with the same dimensions as J (10×6 in this case).

$$J = U\Sigma V \quad [4]$$

The diagonal values of ∑ are ordered in decreasing order. The rows of V are unitary vectors orthogonal to each other. Therefore, if the inputs vary proportionally to the 6th of these vectors:

$$d\vec{x} = \epsilon\vec{v}_6 \quad [5]$$

the output will change proportionally to the 6th column of the matrix U:

$$d\vec{y} = \sigma_6\epsilon\vec{u}_6 \quad [6]$$

The smaller the value of σ_6, the smaller is the effect of the change of inputs on the measured observable outputs and the lower is the possibility of identifying the inputs from experimental measurements.

In order to identify which inputs can be identified with less accuracy from measured experimental outputs, the columns corresponding to each input in the Jacobian matrix should be removed and the singular values recalculated for the new matrix. The higher is the new lowest singular value of the new matrix, the less the accuracy with which the removed input can be identified in the original system.

The described analysis was performed for each of the reactions in our system leading to the results shown in Fig. S3. Fatty acid oxidation (FAO) appeared to be the most difficult flux to

identify followed by pyruvate carboxylase. As the main focus of this study was to assess FAO, it was necessary to perform experiments with siRNAs silencing FASN and ECHS1 and assuming that the rates of glutaminolysis, pyruvate carboxylase and malic enzyme, were not perturbed by these siRNAs.

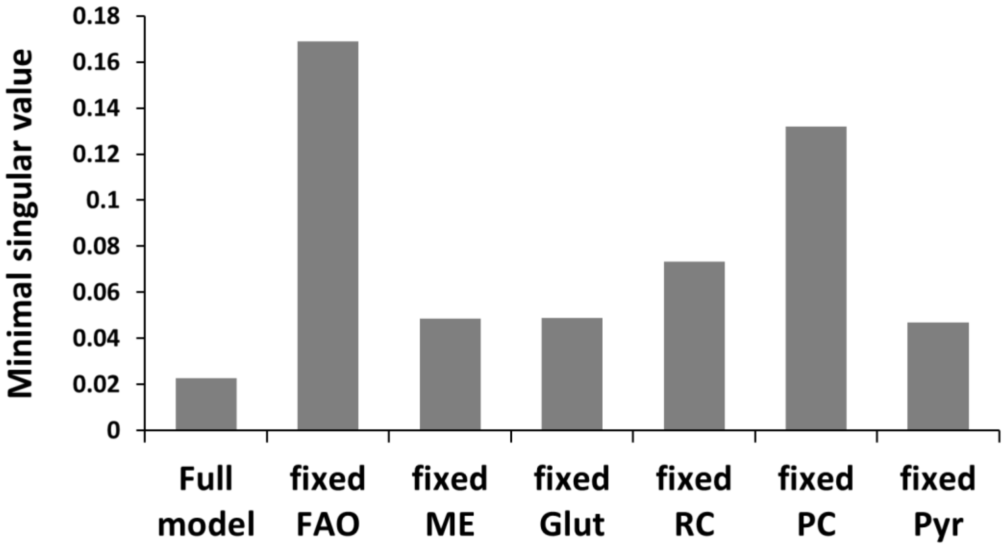

Supplementary Fig. S3 Values of the smallest singular values of the Jacobian matrices of the full system and after fixing (removing it from the Jacobian matrix) each of the independent metabolic reactions independently.

In order to illustrate the identification problem, it is shown (**Supplementary Fig. S4**) how different flux distributions can result in almost in indistinguishable isotopomer distributions.

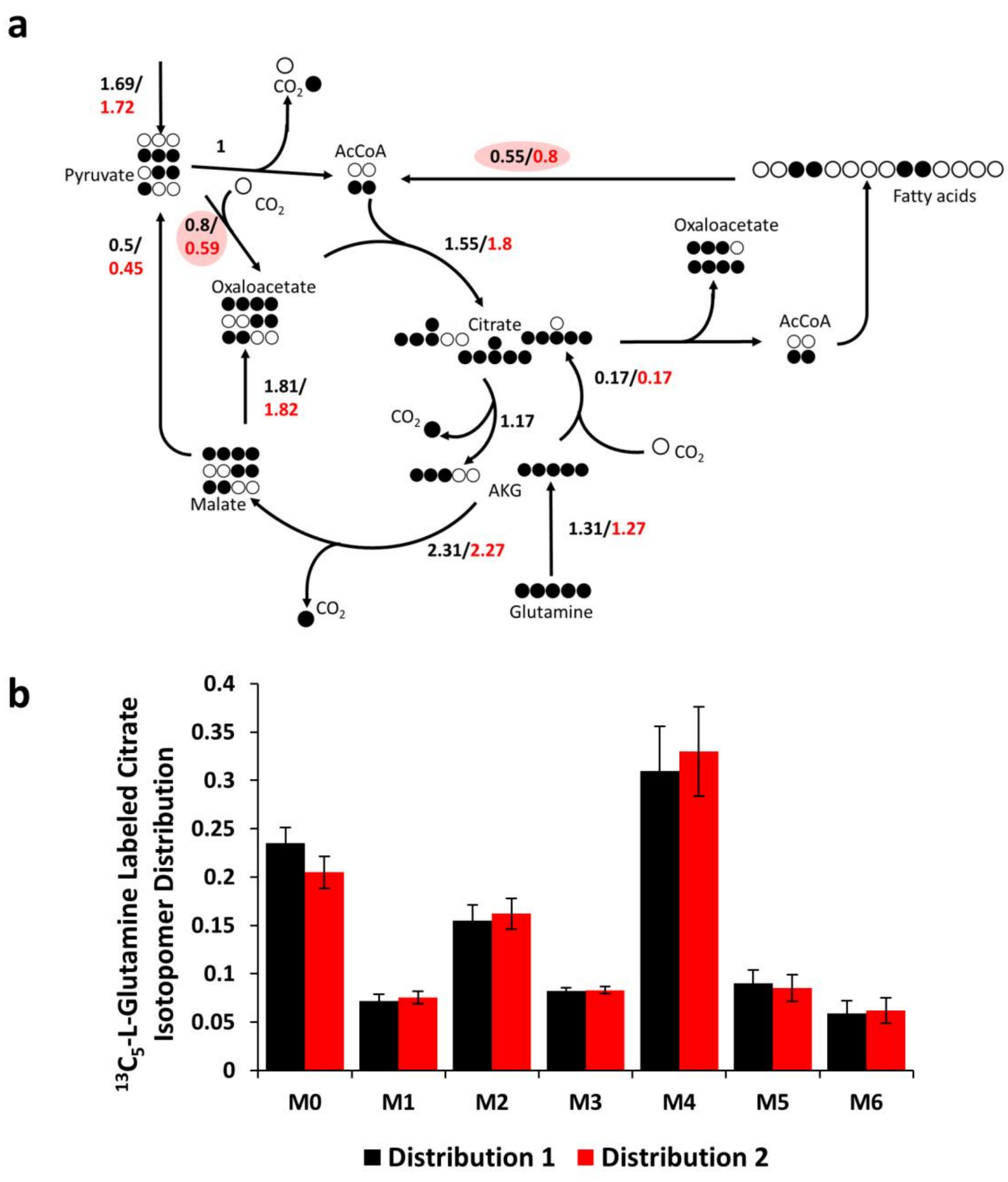

Supplementary Fig. S4 Scheme and citrate labeling patterns of two alternative distributions of metabolic fluxes. **a** Sheme illustrates how flux distributions with a 50% rise in lipid degradation rates can result in almost identical flux distributions. **b** Bar graph illustrates how flux distributions in which FAO differs in 50% from each other lead to labeling patterns non distinguishable experimentally from each other and genetic approaches are necessary to complete metabolic flux analysis. Error bars represent 95% confidence intervals calculated for BCC, they are displayed in order to illustrate the fact that both flux distributions lead to isotopomer distributions that are indistinguishable experimentally.

Calculation of isotopomer distributions using the EMU (elementary metabolic unit) framework

The necessary isotopomer distribution calculations have been performed using a customized python library, which has been uploaded as a python file.

Other data

Flow cytometry and mass spectra raw data have been uploaded in the compressed file raw_data.rar

The python library labels.py, contains an implementation of the EMU method for the network used in this article. The function label takes flux distributions as input and computes isotopomer distributions for citrate and lactate.
